# An Experimental and Numerical Study on the Influence of Helices of Screw Piles Positions on Their Bearing Capacity in Sandy Soils

**DOI:** 10.3390/ma17020525

**Published:** 2024-01-22

**Authors:** Stanislav Simonenko, José Antonio Loya, Marcos Rodriguez-Millan

**Affiliations:** 1 Department of Continuum Mechanics and Structural Analysis, University Carlos III of Madrid, Avda. de la Universidad 30, 28911 Leganés, Madrid, Spain; stanislav.simonenko@gmail.com (S.S.); jloya@ing.uc3m.es (J.A.L.); 2 Department of Mechanical Engineering, University Carlos III of Madrid Avda. de la Universidad 30, 28911 Leganés, Madrid, Spain

**Keywords:** mesh-free modeling, helical piles, experimental tests, bearing capacity

## Abstract

Helical piles became a popular foundation technique, and as a result of environmental restrictions, they have become increasingly widely used. However, due to the high cost of experimentation, the influence of the number of helices and their positions on the pile-bearing capacity has not been sufficiently studied. The present study performed compression and lateral load tests on helical piles of the same diameter but with one, two, and three round helices in known sandy soil. The results from the experiments are compared with those from numerical simulations that use the mesh-free RBF method and the Winkler–Fuss approach to model how the pile and ground interact. The results are generalized to suggest an engineering equation that can predict the best pile configuration in sandy soil.

## 1. Introduction

Screw (or helical) piles are a popular foundation technique that consists of screwing metallic pipes (shafts) into the ground with one or several round helices welded on them. Invented in the 1830s, they have become increasingly popular because of their ecological neutrality and reasonable cost compared to other foundation techniques. Compared with other foundation types, screw piles have many benefits including easy installation, low equipment requirements, removability, reusability, minimal noise and vibration during installation, and cost-effectiveness [1,2].

Screw piles may also be established without excavation or concrete pouring [1]. The geometry of the central shaft has also been discussed in the literature, with most of the designs being based on a circular or square section [3]. The arrangement of the helices, along with the number of them, is also included. Mittal and Mukherjee concluded that increasing the number of helices also increases the ultimate resistance under a compressive load [4]. Shao et al. [5] examined the optimization of inter-helix spacing for helical piles. The researchers conducted a comparative analysis of two theoretical methodologies using existing data from the literature, and also examined the suitability of these methods. The suggested approach was verified by conducting a centrifuge test and using the finite element method approach (FEM) on helical piles in Congleton HST95 sand. They found that the Meyerhof pile foundation theory is inadequate for assessing the ideal inter-helix spacing because of its more significant upper influence zone and lower impact zone. Using a cavity expansion theory approach, considering the sand’s characteristics and the depth of the pile, yields a more precise assessment of the proper spacing ratio. Based on an analytical solution, the best inter-helix spacing ratio for helical piles in dense Congleton HST95 sand is 2.2. The torque factor, which relies on the type of soil, pile shape, mechanical characteristics of soil, loading direction, and installation depth, may be used to calculate the axial capacity of screw piles [6,7,8].

In 1989, Clemence and Hoyt [9] gave an experimental method to evaluate the bearing capacity of a pile as a function of the torque necessary to install the pile. This method is not suitable on any non-homogenous grounds, and the use of it in practice is prohibited by European norms. However, it is commonly used in the United States and Canada (where 10-year insurance is optional for buildings).

Another critical problem in the dimensioning of screw piles is the prediction of their bearing capacity under lateral loads applied on their top—a typical problem of dimensioning buildings exposed to wind loading or seismic loads. The shear loads of a screw pile rise with the number of helices up to a certain depth, after which they rise gradually [10]. Wang et al. [11] conducted laboratory model tests to investigate the bearing mechanism of screw-shaft piles. They found that a threaded design’s substantial lateral resistance and bearing capacity increased. Ou et al. [12] studied the responses of inclined loaded piles in layered foundations. The results revealed that the elastic modulus ratio between the upper and lower soils, length-diameter ratio, and elastic modulus change ratio of the adjacent soil all significantly influence the inclined loaded pile’s lateral displacement and bending moment.

The dimensioning of screw piles in Europe is carried out by the Eurocode 7. More specifically, the norm NF P 94-262 [13] allows the dimension of the piles after conformity tests, i.e., previous testing of the piles by loads that are maximal for the current project. The norms that define these tests are summarized in Table 1:

In addition to experimental studies and analytical models, the dimensioning of helical piles has been carried out using numerical techniques, implying significant savings in developing experimental studies.

The finite element method (FEM) has been used in most numerical simulations [17]. Typical FEM commercial codes used for modeling are ABAQUS, Plaxis 3D, and MIDAS GTS NX. In these suites, the model parameters are adjusted to replicate the experimental results accurately. The accuracy of these finite element models was evaluated by comparing the results with field experiments or small-scale laboratory-modeled testing, proving an important level of consistency [17]. Helical piles bearing capacity modeling has been widely used in scientific literature [18,19,20].

Karami et al. [21] conducted laboratory experiments to study the axial load-settlement behavior of model helical piles of high-tension steel in soft clay soil. In addition, they employed 3D finite element analysis with ABAQUS. The pile capacity was shown to be considerably affected by the pitch and embedded pile length. The FEM study showed that the highest levels of plastic strain and displacement occur between the pile-tip and the interface, while they are minimal at the borders.

A study by Yang et al. [22] examined the bearing capacity, settlement characteristics, and force characteristics of screw-shaft piles under different loading conditions using both experiments and simulations. Among other findings, they figured out that the side resistance of screw-shaft piles first shows a rise as the length of the threaded section grows, reaching a stable value at an ideal length of roughly 0.44–0.55 times the total length of the pile.

Vignesh and Muthukumar [17] used finite element modeling to analyze group piles’ uplift and lateral behavior in soft clay soil. They investigated the group effect for rectangular, triangular, and square configurations. Results show that pile number, spacing, and failure criteria influence group efficiency. The study suggests an optimal spacing ratio for both tensile- and laterally-loaded group helical piles.

Cerfontaine et al. [23] developed a numerical model using the discrete element method (DEM) to investigate the complex soil behavior during screw pile installation. They showed that maintaining a low particle scaling factor is essential to reproduce the correct mechanism at a low pile advancement ratio. Zhong et al. [24] studied snakeskin-inspired piles using DEM. They found that the snakeskin-inspired pile has a higher shaft resistance than the reference pile, and generates significant soil disturbance due to soil displacement and particle. The shaft resistance and soil disturbance positively correlate with the scale height and negatively correlate with the scale length.

Chen et al. [25] conducted an analysis of soil slopes reinforced with piles of varying locations and lengths. The primary aim was to compute the safety factor using the limit equilibrium method (LEM). The findings showed that the construction of piles supplied several possible sliding surfaces, leading to elevated uncertainty of slope collapses.

Most of these techniques require a high CPU time (a few days or weeks) [17] and commercial code license costs. In the present work, the mesh-free radial basis function (RBF) approach, implemented in a self-developed code for this study, is used to predict the mechanical response of helical piles when subjected to axial compressive and lateral loads.

The RBF approach presents a beneficial and significant alternative for interpolating dispersed data and resolving partial differential equations (PDEs) on irregular domains, such as cable trusses [26]. It can be used for elasto-plasticity modeling [27] and, in general, in problems where a high convergence rate is needed or where the geometry of the computational domain can change during the computation (moving boundary problems) [28,29]. Generally, mesh-free methods based on RBF are widely used to solve geotechnical problems due to the flexibility of the approach [30,31]. Neural networks’ RBF computational schemes are used for solving geotechnical engineering problems [32,33]. Generally, RFB-based methods are ideally suited for obtaining smooth solutions for complex geometries [34,35,36]. These methods are also quite convenient for use in phase transition problems in geotechnics [37].

Implementing these approaches is straightforward for any geometry of the computing domain, as using RBF relies on calculating distances between nodes. Another benefit is the potential for achieving high-order approximation regardless of the distribution of nodes, which simplifies grid manipulations and eases dynamic calculations. A notable limitation of the mesh-free RBF approach is its reliance on the number of nodes, similar to the FEM. The accuracy of modeling increases as the number of nodes increases. However, this can lead to convergence issues caused by ill-conditioning, and from a practical standpoint, it can result in much longer computing times. The duration can be lowered using RBF neural networks or parallel programming techniques.

In this work, numerical simulations are conducted on helical piles with one, two, and three helices positioned along the length of the pile. The numerical predictions achieved are verified with our experimental data, for which we have manufactured and evaluated the behavior of piles in sandy soil, measuring their displacement under a specific load.

## 2. Materials and Methods

The method employed in this study involves using the UC3MLib software v03 (http://geoia.fr, accessed on 18 January 2024)—a calculating software founded on the mesh-free RFB computing approach. This software is employed to calculate the bearing capacity of screw piles inside a soil environment, as described by the geotechnical investigation. After that, the numerical results are checked against the experimental data to determine if the proposed technique can be used to determine diverse types of helical piles with the same diameter but different number and disposition of helices. Next, examining the impact of the helices arrangement on the pile shaft concerning its bearing capacity is advisable.

The failure criterion for our study is based on technical agreement No 3.3/21-1044_V1, issued by the Scientific and Technical Centre of Construction (CSTB) in France. This agreement established a maximum displacement of 10 mm under a load of any direction as the threshold for piling failure.

### 2.1. Experimental Setup

- Piles

**Figure 1:**
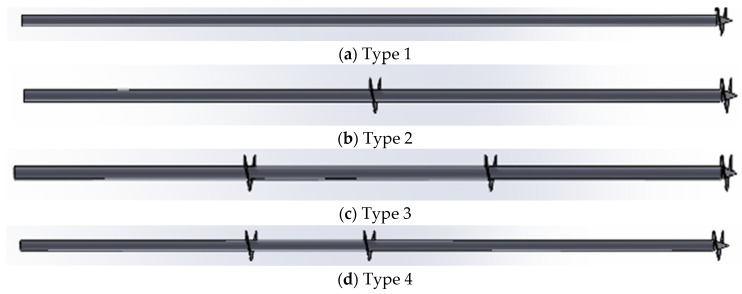
Types of helical piles considered.

Four different types of piles of carbon steel S355 have been used to carry out this study. Every pile has a shaft diameter of 88.9 mm, a length of 6 m, a helix thickness of 5 mm, and a helix diameter of 250 mm. The main difference between the analyzed types is the number of helices used (from one to three) and their position along the pile. Table 2 summarizes the number and positions of the helices, as shown in Figure 1.

- Soil

**Figure 2:**
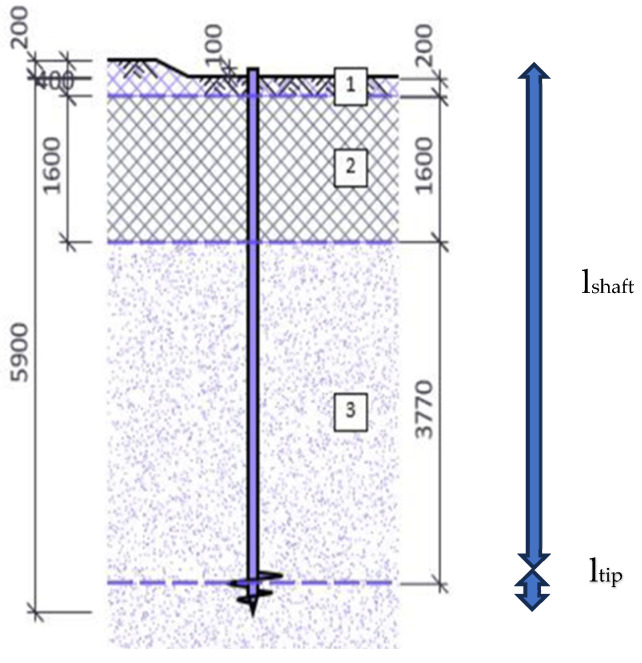
Scheme of position of pile in the horizons of soil. Dimensions in mm.

The experimental tests are carried out in sandy soils in Queven (Western France, Brittany). The GPS coordinates of the location are 47°47′4.127″ N 3°25′15.976″ W. Following the geotechnical study outlined in Table 3, the ground is notable for its moderately compact, reddish-beige granite arena that has a depth of up to 8 m compared to the ground’s surface and becomes compact from 8 m onwards. The pile’s placement in the soil is shown in Figure 2. The piles are screwed in the region up to 5.9 m, and 0.1 m above the surface as is shown in Table 4. According to the design of Figure 2, the pile is immersed 0.2 m in Horizon 1, 1.6 m in Horizon 2, and 3.77 m in Horizon 3.

- Pile preparation and installation

**Figure 3:**
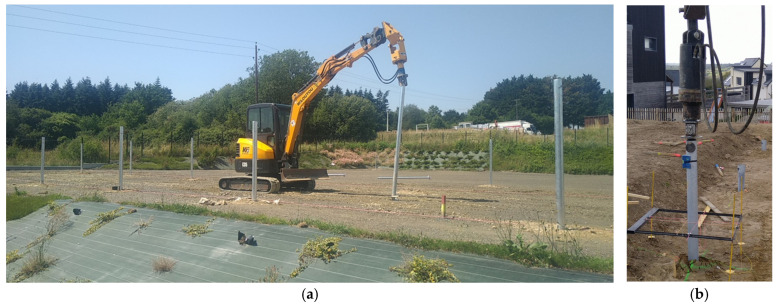
(**a**) Screwing 2-meter-long helical piles with a 3.5-ton excavator in sandy soil as a foundation for a field of solar panels and (**b**) helical pile during installation.

Once dimensioned, the pile is prepared in a workshop, brought to the site, and installed by an excavator or other machinery equipped with a capstan—a low-speed hydraulic engine that screws the pile into the soil. The capstan used for experimentation had a maximal torque of 15 kN·m (Figure 3). During the pile placements, the torque is continuously registered.

Some researchers [38,39,40] relate torque with the bearing capacity, giving an empirical relationship between these two parameters as a linear dependent variable. However, Eurocode 7 does not allow torque with any bearing capacity to be relied on. The explanation is simple: thin and hard horizons in Europe often lay over softer horizons, and anchoring in hard soil does not mean the pile will be stable over a long time period.

According to the guidelines outlined in Eurocode 7, conducting a series of tests on various helical piles at the designated site is necessary. Each test (compression and shear load test), which has a duration exceeding 6 h, contributes significantly to the overall construction expenses, resulting in a substantial cost rise.

- Compression test

**Figure 4:**
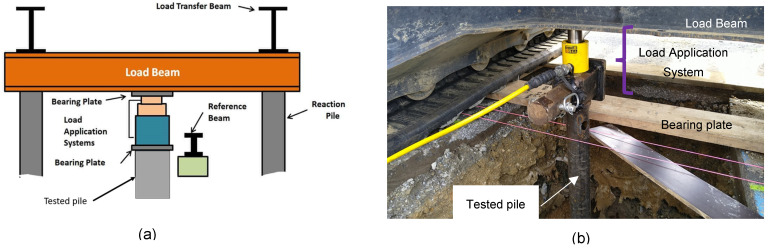
(**a**) Compression test scheme and (**b**) compression load test done on a pile.

The compression test is carried out as follows: once the pile is installed at the projected depth, a load beam (or any other device necessary to compensate for the pile’s reaction to the load) is placed above its head. A load application system—in this case, a precise hydraulic jack—is placed between the pile head and the reaction beam where it is then used to apply a load to the pile head. The reference beam is an independent reference fixed far enough from the reaction beam to not be under any constraint caused by the test, and is used to take measurements of pile displacements. The test scheme is shown in Figure 4.

To test the performance of the piles under different loads, a series of increasing forces were applied, starting from 0.5 tons and adding 1 ton every hour up to 14.5 tons. The vertical movement of the pile concerning the reference beam is recorded at the start and end of each hour-long interval.

Due to the elasticity of the pile, it was necessary to differentiate between the displacement of the pile within the soil and the deformation of the entire pile. To minimize the bending effect, piles were previously filled with C30/40 concrete, which improved inertia and prevented the piles from bending.

- Shear load test

**Figure 5:**
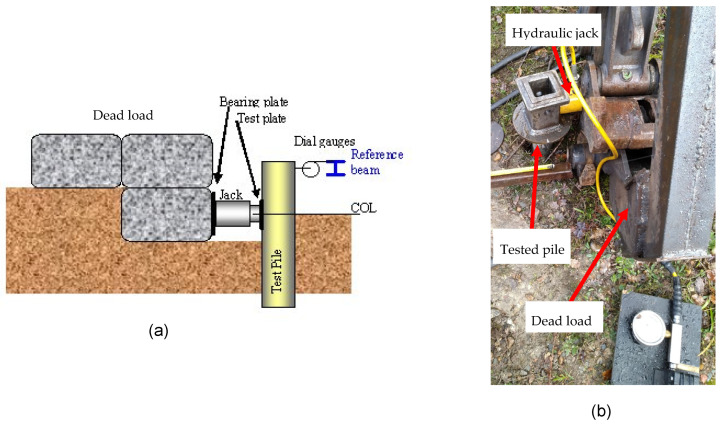
(**a**) Lateral test scheme and (**b**) lateral load test being carried out on a helical pile.

Once the pile is installed in the ground, the lateral loads are created using a precise hydraulic jack system placed between the pile top and a reaction beam or a dead load (often a heavy excavator). The orientation of this system is perpendicular to the pile (Figure 5). The pile is incrementally loaded, like the compression test, with an initial load of 0.5 Tn and subsequent increments of 0.5 Tn until reaching a final load of 9 Tn. 

### 2.2. Development of the Numerical Model

Predicting the pile-bearing capacity through a numerical simulation is essential to determine whether helical piles will suit a particular building site before conducting on-site experiments. Eurocodes require experiments, however, their cost is relatively high and an exact prediction can limit the required tests. The numerical model is performed using an innovative simulation software UC3MLib, using the mesh-free RBF approach to solid modeling. The authors of the current paper developed the software, which is available upon request as freeware. It is a computational library for mesh-free scripting constructed with the C# programming language.

- Helical piles and soil numerical model

**Figure 6:**
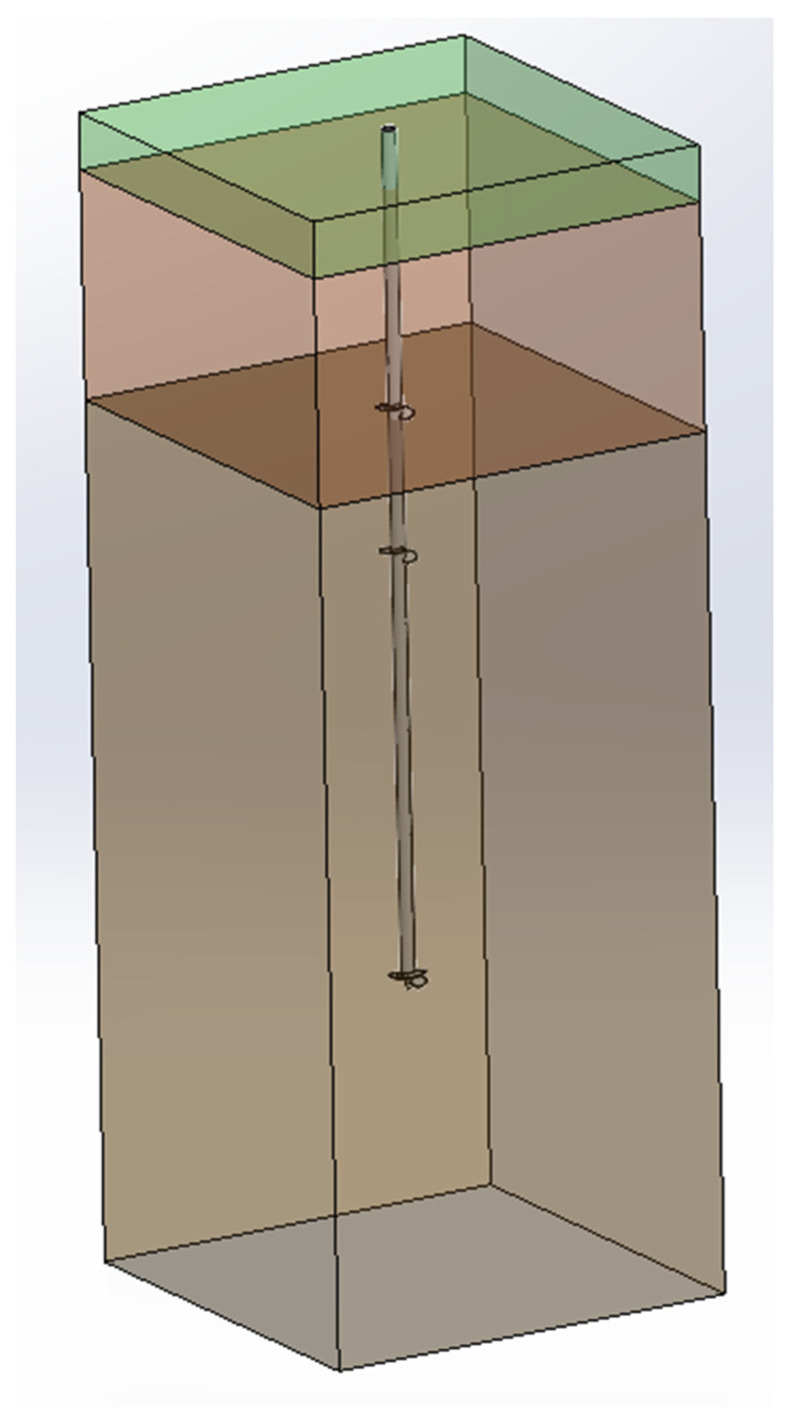
Scheme of domain with a helical pile. Colors represent geotechnical horizons: brown = sandy, orange = alluvial, green = light backfilling.

The following approach models a helical pile in the surrounding soil. An environment modeled is considered as an immersed pile geometry inside a parallelepiped of 6 × 6 m^2^ and 9 m depth (named as “domain”), with a pile centered in it. The domain comprises 10^6^ nodes, composing our grid (Figure 6).

The boundary conditions are constrained at the domain’s bottom and lateral surfaces. Force Q is applied to the top edge of the pile, and the top surface of the domain is free of any constraint.

In the pile and domain, elastic linear behavior is considered. The typical approach to calculating the soil displacement caused by the constraint applied to the pile and distributed to the soil by the pile uses Winkler’s theory, which assimilates the interaction between the pile and surrounding soil as an interaction between a body and a set of linear springs with stiffness K (Winkler coefficient).

The input data consists of a geotechnical analysis, which provides information on the soil types at various depths and the estimated construction loads of the structure that require the use of screw piles for support. Table 3 summarizes the parameters of the soil.

The immersed geometry approach gives K as a continuous function, however, K is a space variable because its value depends on its location within geotechnical horizons. The pile stiffness value is influenced by its depth and position, which differs from that of the soil. This parameter is defined as an immersed geometry parameter for the model. K values are listed in Table 5.

The pile is modeled using carbon steel S355 properties (as piles used for experimental tests). The numerical model of S355 steel is considered to be linearly elastic, with a density of ρ = 7800 kg/m^3^; Young’s modulus is E = 210 GPa; the shear modulus is G = 80 GPa; and the Poisson’s ratio is 0.30. A total of 48,000 nodes are used to model all parts. The displacement of the pile under static load is monitored during the simulation, with the weight raised by 500 kg at each stage. Table 4 summarizes the numerical soil parameters according to Winkler’s theory.

These values are defined following their geotechnical parameters [40,41] in Table 3. Initially, the simulation provides a theoretical bearing capacity for each pile, composed of the bearing capacity of the pile shaft and helix. The bearing capacity accumulates at each geotechnical horizon. This total bearing capacity for traction and compression is calculated for the Type 1 pile (single helix), as shown in Table 5. To obtain the theoretical bearing capacity values, the UC3MLib software calculated the maximal stress of each pile in each soil horizon. Then, $F_{d}$, is the weighted sum computed, Equation (1):

(1) $$F_{d}=\gamma_{c}\left(F_{d0}+F_{df}\right)$$

where $F_{d0}$ is the bearing capacity of the pile helix, $F_{df}$ is the bearing capacity of its shaft and, according to Zhelezkov [42]$\;,\;\gamma_{c}$, is the statistical “work” coefficient given in Table 6.

Table 7, Table 8, Table 9 and Table 10 show how the bearing capacity increases with the number of helices and their placement on the pile shaft. However, this dependence is not linear, and the dependence is studied in the following section.

## 3. Results and Discussion

### 3.1. Experimental Results

**Figure 7:**
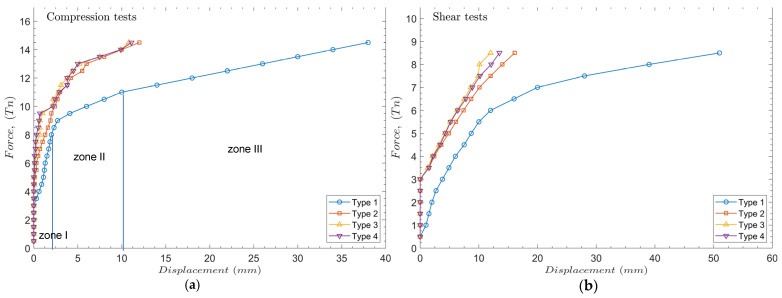
Experimental results. (**a**) Compression tests, (**b**) shear tests.

Figure 7 displays the results of the previously conducted experiments. In Figure 7a, the load-displacement curves can typically be simplified into three distinct regions: initial linear (zone I), transition (zone II), and final linear (zone III), according to Kulhawy [43]. Even if all the deformations remain in the elastic zone, the pile bearing capacity remains in the initial linear zone and other zones match the pile failure.

In compression tests, the type 1 pile helix exhibits the least favorable performance due to its greater displacement of up to 38 mm at 14.5 Tn. However, the other piles show comparable displacements, ranging from 12 to 10.8 mm. A decrease in displacement is observed as the number of helices increases. The analysis of pile types 3 and 4, each consisting of three helices, reveals that the layout of spirals along the pile has a role. An equidistant distribution (type 3) produces smaller displacements (10.8 mm) than a non-homogeneous distribution (type 4) with a higher displacement value (11.1 mm). In the case of the present study, only industrial helical piles are considered, where the helix and the shaft are made of the same type of steel and of similar thickness—a configuration relatively easy to reproduce using industrial tools.

In the case of pile type 1, the displacement is still small (2 mm) until reaching a force of 8.5 Tn. Beyond this threshold, there is a noticeable linear increase in displacement. However, for the rest of the piles, the critical force at which the minimal displacement occurs is around 10 Tn.

Regarding lateral (or shear) tests, the outcomes exhibit comparable curves. Nevertheless, pile type 1 shows a substantial displacement with minimal initial force. The rest of the piles exhibit perfect resistance up to 3 Tn. The one-helical pile (type 1) presents a higher maximum displacement of 63 mm compared to the 18 mm reached in the two-helices pile (type 2), the 14.4 mm of the irregular three-helices pile (Type 4), and the 13.5 mm obtained in the homogeneously dispersed three-helices pile (Type 3). Regarding the compression test, the piles show reduced resistance under lateral loading conditions, indicating that the loads required for the same arbitrary displacement value are lower than in the shear test for the same pile type.

### 3.2. Numerical Results

**Figure 8:**
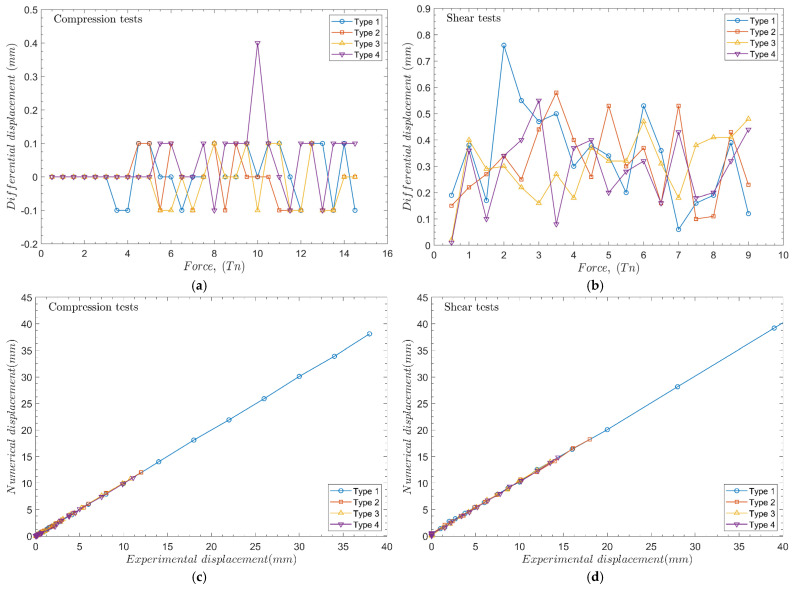
Differential displacement plot between experimental and numerical results. (**a**) Compression tests, (**b**) shear tests. Regression curves of (**c**) compression tests and (**d**) shear tests.

Using the UC3MLib software, curves practically equal to the experimental ones are obtained with errors of 0.1–0.4 mm for the compression test and 0.1–0.75 mm for the lateral load test. These values are adequate compared to pile dimensions and displacement, therefore the model is suitable for helical pile computations. Note that numerical results are not graphically represented because it is invaluable to see the graphical difference. Therefore, a differential displacement plot between the experimental and numerical results is plotted in Figure 8.

Additionally, the average error is still consistent across various pile types, indicating that it is independent of pile geometry. In lateral cases, the distribution of average error exhibits greater variability compared to axial compression load. Furthermore, the average error, ranging from 0.34 to 0.29 mm, reduces as the load increases. On the other hand, the error distribution is scattered and somehow depends on the pile geometry. The error is smaller and distributed more regularly for piles with multiple helices.

The following interpretation can be derived: the total displacement is composed of pile bending and soil deformation for high loads. Under conditions where the load is relatively low (namely, within the elastic domain of the pile or near the plasticity threshold), the only deformation is due to the soil. With increasing load, the total deformation is composed almost entirely of pile shaft bending, and the soil displacement stays almost linear. This means that the error between the test data and simulation decreases.

In Figure 8c,d, a high correlation between experimental and numerical data is obtained with R-squared values of R^2^ = 1 (Type 1), R^2^ (Type 2) = 0.9995, R^2^ = 0.9995 (Type 3), and R^2^ = 0.9991 (Type 4) for the compression tests. For the lateral tests, an R-squared value of R^2^ = 0.9999 (Type 1), R^2^ = 0.9994 (Type 2), R^2^ = 0.9996 (Type 3), and R^2^ = 0.9992 (Type 4) are obtained. With these values obtained, it is proved that the RBF method developed is a helpful tool for predicting the piles’ vertical and lateral displacement.

Once the numerical model has been developed using the RBF method and validated with the experimental results, it is used to investigate the effect of the number of helices and their spacing.

- The influence of the number of helices

Based on the findings from both experimental and numerical analyses, including extra helices in a pile does not significantly enhance its bearing capacity. The increase in capacity is approximately 40% when comparing piles with one and two helices and an average of 10% when comparing piles with two and three helices. However, considering the considerable costs associated with manufacturing and installing piles with additional helices, to obtain a considerable gain in vertical bearing capacity of a helical pile under an axial load, adding a second helix for a 50% vertical bearing capacity increase and two extra equally spaced helices for a gain of 40% of horizontal bearing capacity is a reasonable compromise.

**Figure 9:**
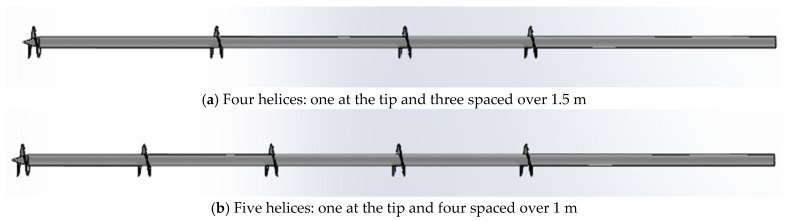
Distribution of the pile with (**a**) four and (**b**) five helices.

Our results differ from the conclusions of Shao et al. [5]. They numerically analyzed the influence of the helices spacing on the bearing capacity of the pile in sandy soils. However, we conducted a numerical simulation of a pile with four and five helices, depicted in Figure 9, which yielded the following results compared to the pile with double helices.

**Figure 10:**
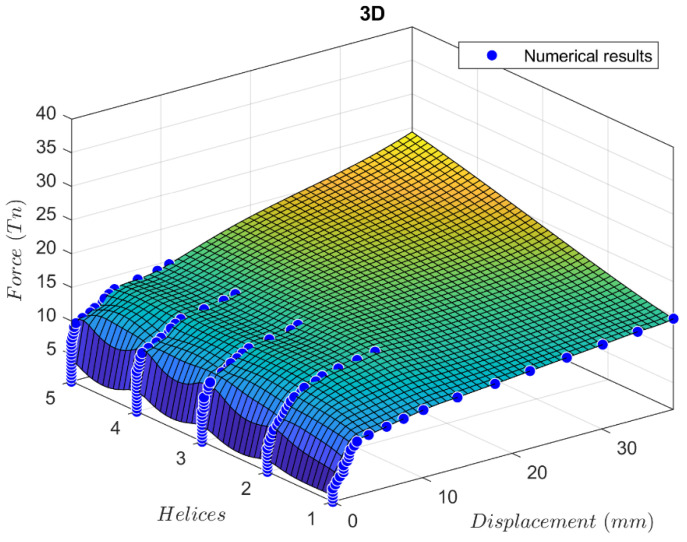
3D representation of load capacity with displacement and number of helices.

The numerical results of the force-displacement curve comparison from one to five helices are shown in Figure 10. For piles with two or more helices, it is shown that the increase in bearing capacity is quite negligible. Therefore, it can be concluded that selecting three helices for a six-meter pile in sand strikes an acceptable compromise between achieving optimal bearing capacity and minimizing manufacturing expenses.

The behavior of the pile, when more than three helices are included, can be attributed to two factors: the destabilization of the ground due to its non-homogeneity on one side, and the three possible states that a loaded pile can be in.

In the initial stage of small loads, most of the load is supported by the lateral section of the pile shaft through pure friction. In the second state, there is a buildup of non-reversible shear deformations. The frictional resistance on the sides of the pile decreases to its lowest values, particularly for weak soil layers. Additionally, there is a redistribution of forces from the main body of the pile to its base. In the third stage, when the loads are close to their maximum, the primary source of the pile’s work is the soil resistance near the pile’s tip.

Only the initial stages of pile work are affected by helices positioned on the upper portion of the pile.

As previously mentioned, the force-displacement plots can be divided into three regions: initial linear (zone I), transition (zone II), and final linear (zone III), according to Kulhawy [43]. An analysis has been performed to obtain the regression line of zone 3 (F=a·d+b) to analyze the influence of the number of helices. Table 11 shows the linear regression curves.

It seems that parameter “a” has an increasing tendency with the number of helices. However, the most significant parameter is given for the number of helices equal to two. This leads to more tests to verify the influence of the number of helices on the linear part of the F-d curve.

- The influence of the space between helices

Four different distributions are generated to examine the impact of the spacing between helices, focusing on the piles of two and three helices for compression and shear loads. The distributions of each setup are presented in Table 12.

**Figure 11:**
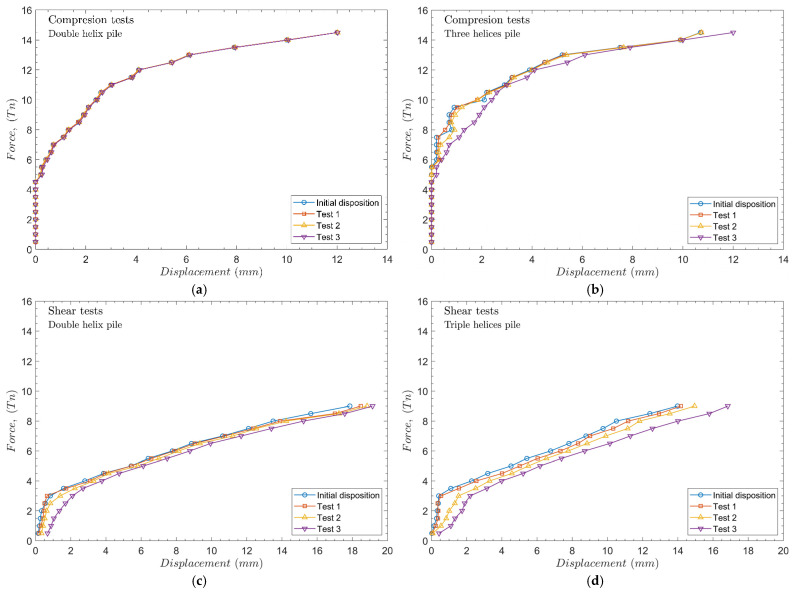
Influence of helices spacing in compression tests with (**a**) double helices and (**b**) triple helices. For lateral tests: (**c**) double helices and (**d**) triple helices.

Figure 11 shows the results of the analysis of the influence of the helices spacing. In the case of two-helices piles shown in Figure 11a, the bearing capacity decreases slightly with the decreasing helix spacing. However, these changes are negligible since the helices remain on the same horizon.

In the case of three-helical piles in compression, the bearing capacity of the pile increases while the spaces between helices become larger according to Shao et al. [38] (Figure 11b). For shorter helices spacing (test 3), the displacement increases (12 mm in the case of test 3). However, for the initial disposition, the displacement is slightly lower (10.7 mm).

Figure 11c,d shows the piles’ lateral bearing capacity results. Similar conclusions are obtained in the case of the application of shear loads. Getting helices closer to each other reduces the bearing capacity of the pile, however, fewer displacements are obtained with a double helix than a triple helix pile for the same load.

Some general considerations drawn from the results are that the pile with several helices works as a ground drill, mostly in non-homogeneous soils. The drill’s rotation destroys natural connections in the ground, decreasing the Winkler stiffness and subsequently reducing its bearing capacity. Nevertheless, if more than one helix is added, the contact area between the pile and the ground increases and the pile becomes embedded in the anchoring horizon (patella-type for a single helix, embedding for a multi-helix pile).

Enlarging the space between helices does not damage the soil during installation and does not cause interference between ground components contained between the helices.

The optimal relationship between the pile bearing capacity and the manufacturing cost, as well as the optimized necessary installation torque, leads us to determine the most efficient configuration of the pile.

## 4. Conclusions

In this work, a numerical model based on the RBF method has been developed to predict piles subjected to compression and lateral loads according to the norm NF P 94-262 of Eurocode 7. The numerical model implemented in a freeware code is validated with the experimental tests achieved in natural soil conditions, showing a good prediction in compression and shear conditions. Therefore, it is a handy tool for manufacturers.

In addition, with the numerical model, an analysis of the influence of the number of helices along the pile and the influence of the distance between helices have been performed. The findings are:

- The proposed numerical model performed by the RBF method performs accurate predictions and can be an alternative to other modes of calculations, such as FEM.
- The load-bearing capacity of piles with two or more helices exhibit minor improvements.
- The optimal distance between helices is the maximum possible value, which enables their distribution within the anchoring horizon.
- Expanding the space between helices does not destroy the soil or interfere with ground parts during installation.
- Increasing the number of helices (>3) does not significantly enhance bearing capacity.
- Only the initial stages of pile work are affected by helices positioned on the upper portion of the pile.

It can be concluded that using three helices for a six meter pile in sand achieves a satisfactory balance between maximizing load-bearing capacity and decreasing production costs.

## Figures and Tables

**Table 1:** European norms governing screw piles tests.

Norm	Experiment Type
NF P 94-150-2 [14]	Traction
EN ISO 22477-1 [15]	Compression
NF P 94-151 [16]	Shear load

**Table 2:** Geometry of tested helical piles.

	Type 1	Type 2	Type 3	Type 4
Number and positions of helices	1 at the tip	1 at the tip 1 at 3 m from the tip	1 at the tip 1 at 2 m from the tip 1 at 4 m from the tip	1 at the tip 1 at 3 m from the tip 1 at 4 m from the tip

**Table 3:** Horizons composing soils and their parameters.

Horizon	Type	Density (T/m^3^)	Cohesion (T/m^2^)	Sr (Humidity Coefficient)	Internal Friction Angle φ (°)	Description	Depth (m)
1	Light backfilling	1.90	0	-	-	Artificial non-compacted backfilling	From 0 to 0.2 m
2	Alluvial backfilling	1.65	0	-	-	Old peat (organic rests)	From 0.2 to 1.8 m
3	Sandy horizon	1.85	0	0.65	28	Reddish-beige granite arena	From 1.8 to 8 m (depth of geotechnical study)

**Table 4:** Pile placement in soil. Data in mm.

Pile overall length, l_p_	6000
Installation depth, Hp	5900
Excavation depth before pile installation (in the Horizon 1), h_e_	100
Length of the free shaft (without considering the tip with first helix), l_shaft_	5670
Length of the pile tip with the helix, l_tip_	330

**Table 5:** Geotechnical horizons for soils and their parameters for modeling.

Horizon	Type	Density (T/m^3^)	Thickness (m)	Winkler Coefficient K (kg/cm^2^)
1	Light backfilling	1.90	0.4	0
2	Alluvial backfilling	1.65	1.6	5.80
3	Sandy horizon	1.85	6.4	10.70

**Table 6:** Work coefficient $\gamma_{c}$ for piles in different soil types [42].

Soil Type	$\mathrm{Work}\;\mathrm{Coefficient}\;\gamma_{c}$ for Load Types:		
	Compression	Traction (Pulling)	Changing Sign
1. Clays and loams:			
(a) hard, semi-hard, and hard-plastic	0.8	0.7	0.7
(b) soft-plastic	0.8	0.7	0.6
(c) fluid-plastic	0.7	0.6	0.4
2. Sands and sandy loams:			
(a) low-moisture sands and hard sandy loams	0.8	0.7	0.5
(b) wet sands and plastic sandy loams	0.7	0.6	0.4
(c) water-saturated sands and fluid sandy loams	0.6	0.5	0.3

**Table 7:** Estimated bearing capacity of a single helix pile (Type 1).

Horizon	Pile Depth (m)	Load Direction	Contact Area A, (m^2^)	Bearing Capacity (T)
3	5.9	Compression	0.049	8.87
		Traction	0.043	7.77
Bearing capacity of pile shaft				
Horizon	Pile depth (m)	H (m)	f (T/m^2^)	Bearing capacity (T)
1	0.30	0.20	-	5.53
2	1.20	1.60	-	
3	3.92	3.85	5.36	
Bearing capacity under compression loads, Tn				10.08
Bearing capacity under traction loads, Tn				7.98

**Table 8:** Estimated bearing capacity of a double helix pile (Type 2).

Horizon	Pile Depth (m)	Load Direction	Contact Area A, m^2^	Bearing Capacity, Tn
3	5.9	Compression	0.098	16.49
		Traction	0.086	14.45
Bearing capacity of pile shaft				
Horizon	Pile depth (m)	H (m)	f (T/m^2^)	Bearing capacity, T
1	0.30	0.20	-	5.53
2	1.20	1.60	-	
3	3.92	3.85	5.36	
Bearing capacity under compression loads, Tn				13.94
Bearing capacity under traction loads, Tn				9.98

**Table 9:** Estimated bearing capacity of a triple helix pile (Type 3).

Horizon	Pile Depth (m)	Load Direction	Contact Area A, m^2^	Bearing Capacity, Tnn
2–3	5.8	Compression	0.135	8.87
		Traction	0.043	7.89
Bearing capacity of pile shaft				
Horizon	Pile depth (m)	H (m)	f (T/m^2^)	Bearing capacity, T
1	0.30	0.20	-	5.53
2	1.20	1.60	-	
3	3.92	3.85	5.36	
Bearing capacity under compression loads, Tn				14.11
Bearing capacity under traction loads, Tn				10.14

**Table 10:** Estimated bearing capacity of a quadruple helix pile (Type 4, Figure 1d).

Horizon	Pile Depth (m)	Load Direction	Contact Area A, m^2^	Bearing Capacity, Tn
2–3	5.8	Compression	0.135	9.96
		Traction	0.043	7.96
Bearing capacity of pile shaft				
Horizon	Pile depth (m)	h, m	f, T/m^2^	Bearing capacity, T
1	0.30	0.20	-	5.53
2	1.20	1.60	-	
3	3.92	3.85	5.36	
Bearing capacity under compression loads, Tn				14.22
Bearing capacity under traction loads, Tn				10.18

**Table 11:** Regression analysis.

N° Helices	Regression Line (F-d)	R^2^
1	F=0.1462·d+0.657	0.979
2	F=0.4395·d+0.2319	0.9104
3	F=0.2799·d+0.0806	0.9097
4	F=0.3055·d+0.0572	0.9094
5	F=0.3452·d+0.0295	0.9086

**Table 12:** Distributions of the spacing between helices.

	N° Helices	
	2 Helices	3 Helices
Initial disposition	0 and 4 m	0, 2, and 4 m
Test 1	0 and 2 m	0, 1.5, and 3 m
Test 2	0 and 1.5 m	0, 1, and 2 m
Test 3	0 and 1 m	0, 0.5, and 1 m

## Data Availability

The data that support the findings of this study are available on request from the corresponding author.
